# Evaluation of heart rate measurements in clinical studies: a prospective cohort study in patients with heart disease

**DOI:** 10.1007/s00228-016-2046-9

**Published:** 2016-03-29

**Authors:** Marco Albanese, Michaelis Neofytou, Taoufik Ouarrak, Steffen Schneider, Wolfgang Schöls

**Affiliations:** Herzzentrum Duisburg, Gerrickstr. 21, 47137 Duisburg, Germany; Stiftung Institut für Herzinfarktforschung, Bremserstraße 79 - Haus M, 67063 Ludwigshafen am Rhein, Germany; Regionalspital Surselva, Spitalstr. 6, 7130 Ilanz, Switzerland

**Keywords:** Heart rate variability, Heart rate measurement, Pharmacological intervention studies, Clinical studies, Heart disease

## Abstract

**Purpose:**

The purpose of this study was to evaluate the measurement of heart rate undertaken in clinical studies by (1) assessing the repeatability and reproducibility of heart rate measurements by various methods and under various conditions and (2) determining whether a single heart rate measurement at rest is representative of the circadian and inter-day variation of heart rate.

**Methods:**

Prospective cohort study in 102 patients with various types of heart disease at Duisburg Heart Center, Germany between 2011 and 2012. The heart rate measurements were based on self-assessment, ECG tracings at rest, and bicycle stress ECG in the office as well as 24-h Holter ECG.

**Results:**

Office measurements and self-assessment at rest as well as 24-h Holter ECG and self-assessment at rest are highly correlated, but no correlation between self-assessment and office recordings/24 h recordings under exercise conditions was seen. Coefficient of variability was below 10 % for the self-assessment and for office measurements at rest. There were no differences in coefficient of variability during the day and within the 6 days for self-assessment of heart rate at rest and circadian variation was normal.

**Conclusions:**

At rest heart rate measurements by various methods agree sufficiently and inter-day/circadian variation is adequately represented. Under exercise conditions self-assessment of heart rate is not valuable and use of 24 h Holter as well as stress ECG recordings is necessary. Thus, self-reported heart rate measurements by the patient at rest seem to be reliable, but should be used in clinical studies only for heart rate assessment at rest.

## Introduction

Previous epidemiological studies generated evidence for a correlation between heart rate and risk of cardiovascular disease and hence life expectancy in the general population and in patients with coronary heart disease (CHD) [1–4]. Most of coronary perfusion takes place in diastole, a phase characterized by a reduced wall tension compared to systole. Prolonging diastole should improve coronary perfusion and might thus decrease CHD morbidity and mortality [5]. Accordingly, several studies have addressed the effect of heart rate lowering drugs on symptoms and prognosis of patients with heart failure and CHD [6, 7].

In these studies, dose titration of heart rate lowering agents was primarily based on measurements of heart rate in ECG tracings at rest, occasionally on 24-h Holter recordings as well. Some studies simply relied on pulse counts obtained by the patients.

The setting for the ECG recording was not specifically defined or described in the study protocols or other publications [8]. Furthermore, a correlation of ECG derived parameters with “real world” measurements of heart rate by the patient during his daily activities has never been performed. There is also some uncertainty on the variability of heart rate measurements within the day and from day to day. Finally, with respect to pathophysiology, the question arises how well a single heart rate measurement at rest reflects the heart rate pattern during the whole day. The lack of information on all these issues sheds some doubt on the significance of single resting heart rate measurements suggested by respective studies.

We therefore analyzed heart rate measurements obtained with various methods and at various points in time. Our primary objectives were (1) to assess the repeatability and reproducibility of heart rate measurements by various methods and under various conditions and (2) to determine whether a single, casual heart rate measurement at rest is representative of the heart rate pattern during the day and between six consecutive days. Furthermore, data collected might shed light on the issue, whether self-reported heart rate measurements by the patient are reliable and, therefore, can be used for heart rate assessment in clinical studies.

## Methods

### Study patients and data collection

This investigation was a single center, prospective cohort study in 102 patients aged 42–80 years with various types of heart disease treated according to ESC and National Guidelines [9, 10]. Patient screening was done in the hospital and data sampling was continued upon discharge from the hospital. Independent of the underlying heart disease, eligible patients had an indication for heart rate lowering therapy (CAD and heart failure) and received all drugs according to standard recommendations. The focus was, however, not on specific therapies, but on the assessment of heart rate under various conditions. Heart rate was determined according to protocol by the patient himself, and by study personnel blinded for the patient measurements, based on resting ECGs, Holter recordings, and exercise test ECG tracings. For the complete protocol, please refer to the online-publication.

### Definitions

Variability in heart rate during the day and day to day variability were analyzed and compared to the heart rate recorded by the ECG at rest in the clinic (office) based on coefficient of variability, repeatability, and reproducibility. Change and reproducibility provide values of the upper limits for the variation with high probability (that is for the difference between repeated measurements). In order to compare self-assessments and the heart rate recorded by the ECG at rest in the clinic, Holter recordings and stress ECG, heart rates agreement was used and defined as a deviation of less than 10 beats/min from mean. For the visual analysis of the 24-h heart rate pattern, we defined a tachycardia period as the duration in time of heart rates deviation of more than 10 beats/min from mean.

### Data analysis

Measurements recorded during the ambulatory period were stored on a personal computer and screened for artifacts as defined by previously described criteria [11]. Only recordings with less than 20 % error measurements were accepted for evaluation. Regarding self-assessment, only daytime values were available.

### Statistical analysis

Categorical variables are described by using percentages and absolute numbers, continuous variables by using mean, standard deviation, and range. The Brevais-Pearson coefficient was used for correlations, and reproducibility was assessed using the Bland-Altman approach [12]. Change between duplicate recordings was calculated by subtracting the first from the second recording. A comparison between baseline and repeat recording was done by using the *t* test. Consistency was obtained by calculating the difference between baseline and repeat recordings, disregarding the sign of the difference.

Repeatability was defined as twice the standard deviation of the changes between two repeated recordings. To compare the reproducibility of different variables, the maximal biological variation (MBV) of the variables was calculated (twice the standard deviation of the between-measurement differences, divided by four times the standard deviation of the two duplicate recordings) [13]. All statistical calculation was performed using SAS 9.3.

## Results

### Clinical and treatment characteristics

The clinical characteristics of the patient population are shown in Table 1. The proportion of patients with hyperlipidemia, hypertension, smoking habits, peripheral arterial obstructive disease, or diabetes mellitus reflected a typical population with heart disease. Almost all were on heart rate lowering medication and most of them had only a slight reduction in ejection fraction. Most performed moderate physical activity in terms of exercise intensity defined by the MET concept at some point of the day. Nearly half of the patients exposed themselves to heavy physical activity striving for cardiovascular fitness and the heart frequency range observed in this study is therefore comparable to that one of a normal, healthy population of the same age range. Exercise levels of peak physical activities at home and during bicycle stress ECG test expressed in MET were comparable in 72 % of patients (data not shown).

**Table 1:** Base line characteristics of patients included in study

Demographics	
Total, %	100 (102)
Female, %	29.4 (30/102)
Age, (years) mean(±SD):	63.2 (±10.43)
Age < 50, %	10.8 (11/102)
Age 50–70, %	60.8 (62/102)
Age > 70, %	28.4 (29/102)
Clinical presentation	
BMI, kg/m^2^ mean (±SD):	27.5 (±3.6)
BMI > 30, %	19.6 (20/102)
Medical history^❋^	
Coronary heart disease, %	75.2 (76/101)
Hypertension, %	91.1 (91/101)
Hypercholesterolemia, %	54.5 (55/101)
Diabetes mellitus, %	12.9 (13/101)
AF, %	14.9 (15/101)
Ejection fraction	
Slight reduction (EF 40–60 %), %	27.7 (28/101)
Moderate reduction (EF 30–40 %), %	8.9 (9/101)
Severe reduction (EF < 30 %), %	3.9 (4/101)
Contraindication to ß-Blockade, %	6.9 (7/101)
Medication^❋^	
Heart rate lowering agents, %	99.0 (101/102)
Diuretics, %	54.9 (56/102)
Antihypertensive medication, %	77.5 (79/102)
Statins, %	75.5 (77/102)
Inhibition of platelet function and anticoagulation,%	95.1 (97/102)
Thyroid hormones	8.8(9/102)
Classification of physical activity in terms of exercise intensity^❋^	
Light, %	77.5 (79/102)
Moderate, %	92.2 (94/102)
Heavy, %	47.1 (48/102)
Very heavy, %	20.6 (21/102)
Unduly heavy, %	3.9 (4/102)

*BMI* body mass index, *SD* standard deviation, *AF* atrial fibrillation, *EF* ejection fraction

^❋^Multiple entries possible

According to the protocol, we expected the patients to record six measurements at rest during the day for a total of 6 days and three measurements per day under exercise conditions. For consistency reasons, we used only self-measurement of heart rate with corresponding Holter recordings and office treadmill heart rate measurements for statistical analyses. Therefore, the number of observations read for the analysis of heart at rest assessed by self-assessment was 3559 in total and 3474 were used. Similarly, for the comparison of heart rate under exercise conditions assessed by self-assessment, the number of observations read was 1831 in total and 1620 were used. Heart rates measured under various conditions are reported in Table 2. Mean heart rate at rest was below 70 beats/min. As expected, standard deviations of the mean heart rate were higher under exercise conditions. Correlation coefficients between office measurements and self-assessment at rest as well as 24-h Holter ECG and self-assessment at rest are shown in Table 3. Correlation coefficients between office measurements and self-assessment at rest as well as 24-h Holter ECG and self-assessment at rest were similar and much higher than any correlation under exercise conditions. In fact, looking at the correlation coefficients for exercise measurements, there was only a very weak association between office measurements and those based on self-assessment.

**Table 2:** Mean (SD), minimum, and maximum values of heart rates measured under various conditions

Period	Mean	SD	Range
HR office at rest^a^	67.4	10.1	45–98
HR office treadmill maximum	118.6	19.2	68–164
HR 24 h all values^b^	69.5	8.7	47–95
HR 24 h minimum	51.5	8.3	35–93
HR 24 h maximum	113.5	17.9	80–172
HR self all values^c^	66.6	9.8	48–105
HR self minimum	57.0	11.1	46–96
HR self maximum	88.6	16.1	60–146

*HR* heart rate, *HR office at rest* HR derived ECG recordings at rest in the clinic, *HR 24 h* HR derived from 24-h Holter recording, *HR self* HR derived from self-assessment; *HR office treadmill* HR derived from a bicycle ECG stress tests, *SD* standard deviation expressed as beats/min

^a^Mean of 3 ECG recordings at rest

^b^Recorded mean of 24-h Holter recording

^c^Mean of all values measured by patients

**Table 3:** Mean values (±SD) and correlation coefficients (CC) between values of heart rate measured under various conditions in the whole study population

Period	Mean values (±SD)		CC
HR office at rest	67.4	10.1	0.68
HR self all values	66.6	9.8	
HR office treadmill maximum	118.6	19.2	0.17
HR self maximum	88.6	16.1	
HR 24 h all values	69.5	8.7	0.67
HR self all values	66.6	9.8	
HR 24 h minimum	51.5	8.3	0.57
HR self minimum	57.0	11.1	
HR 24 h maximum	113.5	17.9	0.26
HR self maximum	88.6	16.1	

*HR* heart rate, *HR office at rest* HR derived ECG recordings at rest in the clinic, *HR 24 h* HR derived from 24-h Holter recording, *HR self* HR derived from self-assessment, *HR office treadmill* HR derived from a bicycle ECG stress tests

Change, consistency, repeatability, mean biological variability (MBV), and coefficient of variability for heart rate measured at rest in the office and with self-assessment are reported in Table 4. Consistency and coefficient of variability indicated a greater variation for office measurements at rest than for self-assessment. For all measurements, the coefficient of variability was below the acceptable value of 10 % and reproducibility was modest. Within self-assessment data, the change was very small but as would have been expected the MBV was higher. Repeatability was worse for the heart rate variability within a day as well as for the heart rate variability within different days during the 12 o‘clock self-assessment at rest. Looking at heart variability of the self-assessment at rest during the day, there were no differences in coefficient of variability during the day (HR self all day) and within the 6 days of self-assessment analyzing the different time points of measurement except for the midday measurement. Analyzing repeatability indices in subgroups of patients with reduced ejection fraction or with different intensities of physical exercise, these indexes were worse, but with no relevant differences in repeatability (data not shown).

**Table 4:** Reproducibility indexes for heart rate measured at rest in the office and with self-assessment by patients

Period	Change	*p* value	Consistency	Repeatability	MBV (%)	CV
HR office at rest	−2.31	0.0046	7.6	14.5	59	9.3
HR self all values	−0.06	0.4777	5.9	15.1	66	7.4
HR self morning values	−0.09	0.2956	5.5	14.5	72	7.6
HR self midday values	−0.07	0.5296	5.8	16.0	73	8.1
HR self night values	−0.09	0.0499	5.3	13.1	71	7.5

*HR* heart rate, *HR office at rest* HR derived ECG recordings at rest in the clinic, *HR self* HR derived from self-assessment, *MBV* mean biological variability, *CV* coefficient of variability

Due to the study design (only one 24-h Holter recording was performed), we used heart rates agreement defined as a deviation of less than 10 beats/min from mean in order to compare heart rates under the various conditions. Maximum heart rates in Holter recordings during daytime will correspond to exercise in a patient under adequate heart rate controlling medication and minimum heart rate will correspond to rest (Table 2). Therefore, it was possible to compare heart rates under exercise (office stress ECG and Holter recordings) and at rest (ECG at rest in the clinic and Holter recordings) to heart rates of self-assessment (Table 5). Comparing heart rates of office stress ECG vs. heart rates of self-assessment under exercise conditions, only 10 % of the measurements showed an agreement with heart rate determined by self-assessment. The same holds true for the comparison of maximum heart rates documented by the Holter recording during the daytime with heart rates of self-assessment under exercise. In this case, only 9 % of patients showed comparable heart rates to the self-assessment. Comparison of the mean heart rate during the Holter recording with the mean heart rate of the self-assessment showed a better agreement with 80 % of the measurements being comparable to the heart rate determined by self-assessment. Similar results were obtained comparing the minimum heart rate of the Holter recording with the minimum heart rate of the self-assessment with 76 % of the measurements showing an agreement.

**Table 5:** Agreement between self-assessment and Holter recordings and office treadmill heart rate measurements

	Patients with HR comparable to self-assessment
HR office treadmill max	10 %
HR 24 h mean	80 %
HR 24 h min	76 %
HR 24 h max	9 %

Proportion (%) of patients showing comparable heart rates with the self-assessment are depicted

*HR* heart rate, *HR office treadmill* HR derived from a bicycle ECG stress tests, *HR 24 h* HR derived from 24-h Holter recording, *HR self* HR derived from self-assessment

Analyzing the 24-h heart rate pattern for tachycardia periods, we observed the following:

1. 60 % of patients showed tachycardia periods with a duration of less than 25 % of the time recorded (range 0–24.3 %, mean 15.9 %) during the 24 h,
2. 38 % of patients presented tachycardia periods with a duration of 25 to 50 % of the time recorded (range 25.3–44.6 %, mean 32.1 %) during the 24 h and,
3. only 2 % of patients had tachycardia periods with a duration of 50–75 % of the time recorded (range 57.6–63.6 %, mean 60.6 %) during the 24 h. During the sleeping hours, the registered tachycardia periods encompassed 12.4 % of the time recorded (range 0–52.2 %).

The study included 15 patients with atrial fibrillation. All of them were already diagnosed with atrial fibrillation and no newly diagnosed atrial fibrillation was detected. Twenty percent (3/15) presented with permanent fibrillation and 80 % (12/15) with paroxysmal fibrillation. In the patients with paroxysmal fibrillation, 9/12 patients showed sinus rhythm, 2/12 patients had permanent fibrillation, and 1/12 patients showed only short phases of atrial fibrillation on 24-h Holter recordings. Analyzing the 24-h heart rate pattern for tachycardia periods, we observed the following: (1) 7/15 patients showed tachycardia periods with a duration of less than 25 % of the time recorded (range 7.5–23.6 %, mean 15.5 %) during the 24 h, (2) 7/15 patients presented tachycardia periods with a duration of 25 to 50 % of the time recorded (range 27.2–37.0 %, mean 34.8 %) during the 24 h, and (3) only 1/15 patients had tachycardia periods with a duration of 50–75 % of the time recorded (range 56.7 %) during the 24 h. In the subgroup of the three patients with permanent atrial fibrillation 2/3 patients showed tachycardia periods with a duration of less than 25 % of the time recorded and 1/3 patients tachycardia periods with a duration of 25 to 50 % of the time recorded. In the subgroup of patients with paroxysmal atrial fibrillation, 6/12 patients showed tachycardia periods with a duration of less than 25 % of the time recorded and 5/12 patients tachycardia periods with a duration of 25 to 50 % of the time recorded and 1/12 patients had tachycardia periods with a duration of 50–75 % of the time recorded.

## Discussion

### Key findings

We hypothesized that the measurement of heart rate undertaken in clinical studies was not representative of (1) the circadian and inter-day variation and (2) of the exercise-induced variation of heart rate during the patient’s everyday life.

Our hypothesis was only partially confirmed and we could show that the exercise-induced variation of heart rate during the patient’s everyday life is not adequately represented in clinical studies. Instead self-reported heart rate measurements at rest represent adequately the inter-day and circadian variation of heart rate. Self-reported heart rate measurements by the patient at rest are thus reliable, but can be used in clinical studies only for heart rate assessment at rest.

### Study strength and limitations

Major part of this study is a measurement method comparison. The statistical aspects of measurement method comparisons have been thoroughly discussed by Altman and Bland [12, 14]. According to their findings, the comparison of mean values by significance testing is inappropriate because a nonsignificant result might occur as result of both a large measurement error and agreement on the average. The calculation of the correlation coefficient is another often misused method because it is not a measure of agreement, but of association. For this reason, we used the coefficient of variability that has the advantage of indicating the relation between the magnitude of observations and their variations. In addition, the use of the coefficient of variation allows for comparison between different methods and different groups of subjects.

Next, defining what is the normal resting heart rate and what is an acceptable deviation is troublesome. We based our decision on the following studies. First, in a study on resting heart rate in Diabetics with altered autonomic function, a standard deviation (SD) of to 10 beats/min was noticed in the healthy comparison group [15]. In a much larger study by Erikssen and Rodahl in 2014 apparently healthy middle-aged men aged 40–59 years, the mean heart rate at rest was 61 beats/min and the observed SD was 9.7 beats/min [16]. In this study, self-assessment was compared to auscultation and resting ECGs. In another publication by Palatini et al. [11] on the reproducibility of heart rate measurement in the clinic and with 24 h intermittent recorders, a SD of up to 9.6 beats/min was accepted. In one of the larger pharmacological intervention studies—the BEAUTIFUL study—a SD of 8.7 beats/min from mean was reported [5, 7]. Based on these studies, we considered a deviation of more than 10 beats/min from mean to be relevant.

Another limit of this study is the relatively small number of patients included in the study, but this is counterbalanced by the high number of observations used. Furthermore, we did not have a normal control group. However, this is not interfering with the objective of this study focused on the evaluation of heart rate measurement undertaken in clinical studies.

### Comparison with other studies and interpretation of results

As to our knowledge, there are no published studies on this argument and therefore comparison with the results of others may be only indirect and limited.

In healthy individuals, there is a circadian pattern of heart rate with a peak heart rate around 10 am, and a gradual decline during the night with a nadir just prior to awakening [17]. Disease states such as congestive heart failure with alterations in autonomic tone and medications can have an effect on circadian rhythms [18]. The healthy controls in two studies on abnormalities in ambulatory 24 h heart rate in diabetes and in inappropriate sinus tachycardia showed tachycardia periods spanning 29 and 42 % of the registered 24 h, respectively. No tachycardia periods occurred during the sleeping hours [19, 20]. This heart rate pattern was not changed in our population on heart rate lowering therapy except for the presence of tachycardia periods during the sleeping hours. Furthermore, there were no differences in coefficient of variability during the day and within the 6 days for self-assessment of heart rate at rest. Due to the low numbers and missing follow-up evaluation of a possible prognostic, significance of these tachycardia periods during the night is not possible. In addition, self-assessment with acquisition of about 36 values per patients as a method of measurement is only slightly superior to three ECG recordings at rest in evaluating resting heart rate. It is noteworthy that the coefficients of variability of both methods are with a value of less than 10 % in an acceptable range. This confirms previous data that heart rate recorded over the 24 h has a better reproducibility than office heart rate and may thus be a better prognostic indicator than traditional measurement of resting heart rate in the hospital setting [11]. A possible reason for the higher statistical correlations in this study with respect to other studies comprising healthy subjects maybe the use of heart rate controlling medication [11].

Furthermore, atrial fibrillation might have an impact on the reproducibility of the various heart rate measurements. According to our results, atrial fibrillation was not a confounding factor due to fact that most patients in this study had only paroxysmal fibrillation with a majority of patients still showing sinus rhythm. In addition, analysis of the 24-h heart rate pattern for tachycardia periods showed that the results of patients with atrial fibrillation were comparable to patients with sinus rhythm.

With regard to heart rate assessment under exercise or stress conditions, self-assessment underestimates the maximum heart rate patients are exposed to. This seems to be supported by the fact that peak exercise levels at home and during bicycle stress ECG were similar in a majority of patients. Thus, from a methodological point of view, bicycle exercise ECG as well as 24-h Holter recordings are more precise in documenting the maximum heart rates reached. Reasons for the above observation might be (1) the time delay between exercise and self-measurement and (2) the fact that the patients may simply have omitted the self-assessment in certain situations.

Our study suggests that there is no pronounced variability of heart rate measurements taken at various time points neither during the day or at different days and, therefore, physiological variation in heart rate does not seem to be a confounding factor in the conduction of pharmacological intervention studies. With regard to the recording of the maximum heart rate under exercise or stress conditions self-assessment of heart rate is not a valuable neither a trustworthy method of investigation. Furthermore, heart rates registered point to relevant tachycardia under exercise or stress conditions. Noticeably in none of the intervention studies published so far, the maximum heart rate on heart rate limiting medication was documented. Based on the results of this study, we would recommend future studies to address the question, whether the extent of the heart rate limiting effect under exercise or stress conditions influences the end points such as cardiovascular morbidity and mortality.

## Conclusions

Dose titration of heart rate lowering agents in pharmacological intervention studies is actually based mostly on measurements of heart rate in ECG tracings at rest. In our study, heart rate measurements at rest in the office and based on self-assessment are comparable and reproducible. Physiological variation in heart rate does not seem to be a confounding factor in the conduction of pharmacological intervention studies. But under exercise conditions, heart rate measurements taken in the office and by 24-h Holter monitoring do not agree in a sufficient manor with measurements based on self-assessment.

Consequently, self-assessment of heart rate provides the same information as conventional methods of measurement at rest. Under exercise conditions, the routine use of 24-h Holter as well as stress ECG recordings is helpful for a more complete data acquisition. Therefore, (1) self-reported heart rate measurements by the patient seem to be reliable only at rest and, therefore, should be used in clinical studies only for heart rate assessment at rest and (2) it seems reasonable to propose a standardized operating procedure for heart rate measurements in clinical studies comprising ECG tracings at rest during every visit (a minimum of three), three treadmill ECG, and three 24-h Holter recordings. Such common standardized operating procedure may enable the scientific community to compare more easily clinical studies without being limited by different methodological designs.
